# How Do Determiners of Job Performance Matter During COVID-19? The Conservation of Resource Theory

**DOI:** 10.3389/fpsyg.2021.774552

**Published:** 2022-04-14

**Authors:** Wen-Xuan Zhao, Lijin Shao, Mingjun Zhan, Michael Yao-Ping Peng

**Affiliations:** ^1^School of Economics and Management, Huaiyin Normal University, Huai’an, China; ^2^School of Economics and Management, Fujian College of Water Conservancy and Electric Power, Yonan, China; ^3^School of Economics & Management, Foshan University, Foshan, China

**Keywords:** perceived organizational support, occupation self-efficacy, subjective wellbeing, job performance, COVID-19

## Abstract

During the COVID-19 pandemic, business managers are facing many challenges from a severe challenge. Many organizations have changed their original management mode and organizational behavior to improve employees’ organizational citizenship behavior, thus reducing their sense of anxiety and incapability. Thereinto, job performance of the employees also affects the growth and development of the organization. To explore how to fragment employees’ positive psychology and job performance, this study discusses the influence on employees’ subjective wellbeing and job performance from relevant factors at the organizational and individual levels. Also, to explore the influence of organizational support and occupation self-efficacy on job performance and the mediating role of subjective wellbeing during COVID-19, a total of 618 valid questionnaires were collected from all walks of life in 2020. Hypotheses were tested by structural equation modeling and Bootstrap technology. The results show that: (1) Professional self-efficacy and subjective wellbeing have a significant positive impact on job performance; (2) Subjective wellbeing plays a complete mediating role between organizational support and job performance, and subjective wellbeing plays a partial mediating role between professional self-efficacy and job performance; (3) Compared with the sense of organizational support, the positive effect of self-efficacy on job performance is more significant.

## Introduction

The outbreak and spread of the COVID-19 at the end of 2019 is a major crisis event that the world faces, which makes all organizations encounter unprecedented challenges in a sudden. External crisis events often give rise to increased uncertainty for staff and pose a direct threat to the performance and survival capability of the organization (Carnevale and Hatak, 2020). For instance, the face-to-face communication for a large number of employees in the information service and banking industries has had to be replaced by the online communication during the pandemic, giving rise to the reduction of work efficiency and thus job performance. The low work efficiency often has a negative effect on the business efficiency and profitability of the entire organization, which makes the discussion on employees’ job performance become essential (De Clercq et al., 2019; Lee et al., 2021). Job performance has always been the focus within the organization (Downes et al., 2021). During the pandemic, organizational performance is the key factor to establish the survival capability of the organization. Thereby, studying the job performance of employees in enterprises under the circumstances of COVID-19 is quite necessary.

Since the job performance of employees is largely affected by the resources owned by them (Hobfoll, 2001; Buchwald, 2010; Barnett et al., 2012), employees often face the loss of internal and external resources in crisis events (Jemini-Gashi et al., 2019). The job demands-resources model usually emphasizes (Bakker et al., 2005) that both external and internal resources can relieve the work stress for employees and promote their performance at work. Thus, to maintain the employees performance during a global crisis, it is necessary to provide support for the external resources at the organizational level and motivate employees to improve the internal individual resources.

In regard to the discussion on the effect of external resources on job performance, there are relatively much more studies on the effect of perceived organizational support on job performance (Lee et al., 2021), for employees are inevitably linked to all kinds of organizational support in the daily work. As a kind of external work resource, the organizational support includes not only support of intimacy and support of respect, but also instrumental support such as information, instrument and equipment, etc. (McMillan, 1997; Akgunduz et al., 2018). One of the most prominent features of the pandemic during 2020 lies in the increasing number of people who work at home. Since work-at-home is a new form of work that is different from the traditional one, the organizational support for employees is especially important (Pahlevan Sharif et al., 2018). Because if there is no instrumental support provided by the organization, it is quite different for employees to carry out work, and they are more eager to have emotional support from the organization during this period. Thus, the organizational support during the pandemic plays a significant role in the job performance of employees (Akgunduz et al., 2018; Meyers et al., 2019). As for the discussion on the effect of internal resources on job performance, there are many pieces of research in terms of psychological capital (Luthans et al., 2005; Choi et al., 2019). Individual psychological capital is generally more important than human capital and social capital, which is a core psychological factor to promote individual development and improve job performance, as well as the key to enhance organizational competitiveness (Luthans et al., 2004; Lin and Si, 2010). In a general way, psychological capital contains four dimensions of self-efficacy, hope, optimism and restoring force, among which self-efficacy is the individual internal resource that has the most significant effect on job performance (De Clercq et al., 2019; Downes et al., 2021). Besides, previous research report that self-efficacy plays a vital role in improving individual performance in the face of dilemma (Amini and Noroozi, 2018; Jemini-Gashi et al., 2019; Liguori et al., 2019). Meanwhile, there is research indicating that compared with general self-efficacy, occupational self-efficacy is more suitable for studying employees in enterprises (Rigotti et al., 2008). Thus, in this paper, it is considered that the occupational self-efficacy of employees during the pandemic is an important kind of internal resource that influences job performance.

According to conservation of resource theory, both perceived organizational support and occupational self-efficacy belong to resources beneficial to individual development (Hobfoll, 2002; Akgunduz et al., 2018). Through acquiring these two kinds of resources, the subjective wellbeing of employees can be improved and the stress of employees can be kept down (Liguori et al., 2019; Meyers et al., 2019). In the organizational context, subjective wellbeing often plays a significant positive effect on job performance (Stallman et al., 2018), and the employees who have perceived of wellbeing tend to have a better performance, and relatively speaking, they are more inclined to make achievements and gain performance (Lyubomirsky et al., 2005; Thompson et al., 2017). More importantly, when faced with crises such as the pandemic, the subjective wellbeing of employees provides great theoretical and practical significance to assist enterprises in overcoming difficulties and achieving the resumption of work and production (Carnevale and Hatak, 2020). Hence, the subjective wellbeing is taken as the mediating variable in this research for further exploring the intrinsic influencing mechanism of perceived organizational support and occupational self-efficacy on job performance during the pandemic.

Based on the above analysis, the research takes the conservation of resource theory as the research perspective, aims to explore the relationships among perceived organizational support, occupational self-efficacy, subjective wellbeing, and job performance during the pandemic. Thus, the aims of this study aims are: (1) from the organizational level and individual level, explore the effect that perceived organizational support and occupational self-efficacy which are regarded as external and internal resources play on subjective wellbeing and job performance during the pandemic. (2) Whether the subjective wellbeing of employees during the pandemic plays a mediating role in the effect on job performance brought by perceived organizational support and occupational self-efficacy. The innovational aspects are: (1) it is empirically examined that internal and external resources play a simultaneous effect on job performance, and comparison and analysis are made to reveal the influence level that internal and external resources bring to job performance. (2) It enriches the research mechanism for job performance in crisis situations, which starts from internal and external resources and conducts via the path of subjective wellbeing, and then have an effect on job performance.

## Literature Review and Hypotheses Development

### The Conservation of Resource Theory

The conservation of resource theory is often employed to discuss the adjustments and adaption of supply and demand status of individual resources. As indicated by the theory, individuals would take actions to acquire, conserve, protect and cultivate their valued resources in order to achieve a balance between supply and demand of such individual resources (Hobfoll, 1989; Harju et al., 2016; Hobfoll et al., 2018; De Clercq et al., 2019), which is called resource acquisition. On the contrary, resource loss is a key factor that constitutes the stress reaction mechanism. Individuals will feel psychologically uncomfortable when individuals suffer the potential or actual resource losses and face the failure in gaining return on input resources (Bruning and Campion, 2018; Zhang and Parker, 2019). Hobfoll (1989) defined resources as objects (e.g., remuneration for work), conditions (e.g., integrity of management systems), personal characteristics (e.g., personality trait), and energies (including intrinsic energies such as inner feeling, and extrinsic energies such as being recognized and sense of achievement) (Hobfoll et al., 2018). Confronted with work pressure, individuals will react with all their owned resources, and the negative impact of pressure will show up when individuals feel the insufficiency of resources in the case of continuous loss but lack of replenishment of resources (Carnevale et al., 2018; Zhou et al., 2019). On the contrary, individuals will feel less stressed if they are able to properly conserve existing resources and replenish new ones (Kuijpers et al., 2020). Given this, based on the conservation of resource theory, this study aims to discuss whether employees under challenges and risks brought about by the COVID-19 pandemic can improve their positive wellbeing in life and work, and further enhance their job performance when organizations provide effective organizational support and strengthen the employees’ belief in completing tasks. Furthermore, this study will be an enrichment for the conservation of resource theory.

### Subjective Wellbeing

Subjective wellbeing usually refers to a state in which a person is dominated by positive emotions and less pessimistic emotions occur during a certain period (Diener et al., 2009). Some scholars argued that wellbeing is the subjective emotional experience from an individual, which represents people’s demands and values by virtue of real life (Kurtessis et al., 2017; Meyers et al., 2019). The subjective wellbeing of people is a kind of positive psychological feeling perceived by an individual for his or her own existing conditions, which is generated by the combined effect of many elements (Meyers et al., 2019). As for subjective wellbeing, such features like subjectivity, relative stability, and integrity are given prominence. Subjective means making judgments about one’s own life satisfaction and emotional state by individual standards rather than uniform standards (Kurtessis et al., 2017). Relative stability indicates that one individual makes judgments about a long-term life satisfaction and emotional experience rather than a temporary period of state. Integrity refers to the comprehensive judgments made about the indicators of life quality related to one’s own positive emotions, pessimistic emotions, and life satisfaction (Diener, 1984; Lee et al., 2021).

For the past few years, positive psychology has attracted more and more attention from the academic circle. Correspondingly, subjective wellbeing which is viewed as part of the main research content of positive psychology, has gradually become the focus of attention from the academic circle. From aspect of positive psychology, it serves to explore self-psychological adjustment and the macro-consciousness of the individual’s inner self; a sense of evaluating the function of the self in life through public and social norms; and lastly, emotional wellbeing as the individual’s awareness and assessment of the emotional state of self-life (Gillet et al., 2012; Evans et al., 2017). A study by DeNeve indicates that positive strength such as self-efficacy, social support, and extroversion plays a positive effect on individual subjective wellbeing (DeNeve, 1999). Moreover, a study by Diener and Chan (2011) has shown that the improvement of subjective wellbeing contributes to individual physical health, which is also conducive to stimulating their creativity and problem-solving skill, as well as motivating prosocial behaviors and higher level of work engagement (Lyubomirsky et al., 2005). Thus, improving subjective wellbeing may imperceptibly facilitate individual employees to be involved in harder and more intelligent work within the organization.

### The Relationships Among Perceived Organizational Support, Subjective Wellbeing and Job Performance

Perceived organizational support was first proposed by Eisenberger et al. (1986). It is defined as an integral perception from employees that whether the organization values their own performance and cares about their own material benefits (Eisenberger et al., 1986; Akgunduz et al., 2018; Wang et al., 2020). According to the conservation of resource theory, an individual usually needs to depend on various resources for the maintenance of current situations and growth (Kurtessis et al., 2017; Liguori et al., 2019). Thus, an individual not only needs to conserve and utilize the existing internal resources, but also necessarily explores and acquires external resources in need (Lee et al., 2021). As a kind of resource, the support from the organization cannot only provide assistance for employees, but also resolve difficulties during their work, thus contributing to the improvement of job performance during their work. Eisenberger et al. (1986) found that when employees have personal experience of the support, care and recognition from the organization, they will show good job performance. He argued that the organizational support for employees can enhance the dependence of employees on the organization, so as to improve the recognition and expectation for the organizational goals (Meyers et al., 2019). It has been confirmed in the meta-analysis on perceived organizational support conducted by Hurt et al. (2017) that perceived organizational support plays a positive effect on job performance. Aselage and Eisenberger (2003) considered that the perceived organizational support is a positive commitment, and once employees perceive of the support from the organization, they will be dedicated to assisting the organization with its goals. As such, this study proposes the following hypothesis:

*H1: Perceived organizational support plays a positive impact on job performance.*

A study by Diener (2012) has indicated that the social environment in which people exist mainly refers to social support from others and interpersonal relationship with others, which influences the individual evaluation of life and wellbeing perceived by them (Diener, 2012). Based on the conservation of resource theory, when an individual acquires the resource of organizational support, it can contribute to resisting against stress for employees and enhancing the subjective wellbeing (Hobfoll, 1989; Hobfoll et al., 2018). Thereinto, the emotional support for employees from the enterprise is the part of perceived organizational support which plays the most influential role in the subjective wellbeing of employees, and the support of instrument and information is the second most influential (Wang et al., 2020). Now there are many pieces of research that have confirmed the positive correlation between perceived organizational support and subjective wellbeing (Pahlevan Sharif et al., 2018). Caesens et al. (2016) argued that due to the different work experience, the same individual has different perception of perceived organizational support in a few weeks, and it is found that the perceived organizational support in each week plays a positive effect on the subjective wellbeing in each week by influencing the engagement in work. In the research on the relationship between perceived organizational support and subjective wellbeing of Chinese nurses conducted by Yu et al. (2019), it is found that perceived organizational support is a major element influencing subjective wellbeing via hierarchical regression. Thus, this study proposes the following hypothesis:

*H2: Perceived organizational support plays a positive impact on subjective wellbeing.*

When employees feel organizational support, the external resources will be increased, thus making them perceive of wellbeing (Lee et al., 2021). The wellbeing of employees during their work is significant both for enterprises and individuals, because positive emotions can both broaden people’s intelligence, physiological and social resources, and be conserved as a kind of resource for employees to seize job opportunities, face challenges and improve job performance (Kurtessis et al., 2017; Meyers et al., 2019). There is much evidence showing that an employee with wellbeing is more inclined to achieve success in many fields (Yang, 2014; Pahlevan Sharif et al., 2018; Wang et al., 2020). Oswald et al. (2015) confirmed by comparing the experimental group with the control group that the improvement of wellbeing can affect the improvement of production efficiency, indicating a causal relationship between wellbeing and job performance (Huang et al., 2019). Based on the associated data of employers and employees, Bryson et al. (2017) investigated the relationship between subjective wellbeing and job performance of British employees, resulting in a significant correlation between work satisfaction in the workplace and job performance. The mediating role of subjective wellbeing in job performance has also been verified by many scholars. Darvishmotevali and Ali (2020) found from an investigation of hotel staff in Tehran, Iran that subjective wellbeing mediates between organizational support and job performance. Thus, this study proposes the following hypotheses:

*H3: Subjective wellbeing plays a positive impact on job performance.*

*H4: Subjective wellbeing plays a mediating role between perceived organizational support and job performance.*

### The Relationships Among Occupational Self-Efficacy, Subjective Wellbeing and Job Performance

The concept of self-efficacy was initially proposed by Bandura (1977), which refers to an individual’s judgment of his or her capability of engaging in certain activities. Self-efficacy can generally be classified into two types, that is, general self-efficacy and domain-specific self-efficacy (Alisic et al., 2020; Downes et al., 2021). Occupational self-efficacy belongs to domain-specific self-efficacy, which is developed based on self-efficacy, and is generally defined as “the generic terms for a series of behavioral efficacy judgments about occupational adjustments and range of choice during the job selection of individuals” (Lent and Hackett, 1987; De Clercq et al., 2019).

An individual usually owns the capability of perceiving self-efficacy, the self-efficacy perceived by the individual is generally in direct proportion to the efforts paid and endurance (Seo and Ilies, 2009; Downes et al., 2021). Those people who hold a high belief of self-efficacy usually persist in work for longer time, and search for more challenging tasks, eventually leading to higher performance (De Clercq et al., 2019). While testing the construct validity of the occupational self-efficacy scale, Rigotti et al. (2008) found that there is a correlativity between occupational self-efficacy and perceived performance. Chae and Park (2020) conducted an investigation among 140 key employees and their colleagues from 15 industries in South Korea, and found that the personal characteristic of general self-efficacy plays a significant positive effect on job performance. In combination with social cognitive theory and social comparison theory, Downes et al. (2021) suggested that employees with a high level of job-based self-efficacy will set higher task goals and make full use of their capabilities to achieve superior performance. To sum up, it can be expected that employees with high occupational self-efficacy are more inclined to have better job performance. Thus, this study develops hypothesis as follows:

*H5: Occupational self-efficacy plays a positive impact on job performance.*

Self-efficacy usually shows the level of self-confidence for individuals. A high degree of self-efficacy generally represents a high level of self-confidence. Individuals with strong self-confidence are more likely to have positive emotions, which is conducive to enhancing the experience of wellbeing at work (Jemini-Gashi et al., 2019). Currently, there are a number of studies which have confirmed that self-efficacy and subjective wellbeing are positively correlated to a significant extent. For instance, scholars from Israeli have found that prenatal self-efficacy of pregnant women plays a significantly positive effect on postpartum subjective wellbeing (Miri et al., 2016). From the perspective of social acceptance, scholars have explored the relationship mechanism between self-efficacy and subjective wellbeing, and found that self-efficacy plays a significantly positive effect on the subjective wellbeing of individuals.

Being a positive personality trait, self-efficacy is regarded as one of the forms of individual resources (Hobfoll, 2002; Lee et al., 2021). According to the conservation of resource theory, an individual will strive to acquire and maintain valuable resources and turn them into positive results. The gain of resources corresponds to the increase of subjective wellbeing, which indicates the increase of positive emotions and the decrease of negative emotions at work (Hobfoll, 1989). Based on the job demands-resources model, Huang et al. (2019) examines relationship between self-efficacy and wellbeing of teachers in primary schools, and the results show that self-efficacy will have a positive impact on enthusiasm and contentment in wellbeing, but a negative impact on anxiety and depression. When the positive emotions of employees at work are more than the negative emotions, their working enthusiasm will be improved, thus eventually leading to high job performance. On this basis, this study develops hypotheses as follows:

*H6: Occupational self-efficacy plays a positive impact on subjective wellbeing.*

*H7: Subjective wellbeing plays a mediating role between perceived organizational support and job performance.*

**FIGURE 1:**
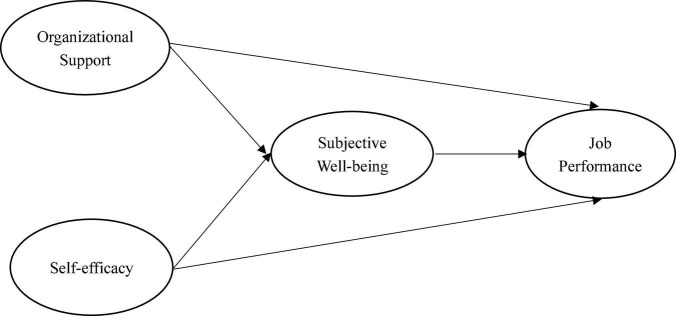
Research framework.

Based on the above hypothesis, this study proposes the following research framework Figure 1.

## Methodology

### Sampling

This study aims to understand the psychological characteristics of frontline staff during major events, especially during the COVID-19 pandemic. It explores the relationship between perceived organizational support, subjective wellbeing and job performance. As there are different quarantine procedures in different countries, and the pandemic plays different influences on people’s psychological characteristics, it is impracticable to take each country as a sample. Thus, purposive sampling is adopted, and several conditions will be established during sampling so as to improve the representativeness of the research samples. First, mainland China, where the pandemic was most severe in the beginning, was selected as the main area for sampling, and the quarantine policy was the strictest. Thus, it is representative to a certain extent. Second, to understand the psychological characteristics of front-line staff, it is necessary to focus on those who actually face customers, and the service industry was adopted as the main industry. Third, while filling the questionnaire, all the samples were already at work, rather than being isolated at home. This study takes the front-line staff in the service industry, excluding the staff in the catering service industry, as the study population in order to accurately collect representative samples. In this study, copies of electronic questionnaire were sent, and 680 copies of questionnaire were collected. 618 copies of valid questionnaire were obtained after excluding invalid ones. The sample background is shown in Table 1.

**TABLE 1:** Demographic characteristics of research samples.

Variable	Sample size	Percentage (%)	Variable	Sample size	Percentage (%)
Gender			Hold a managerial position		
Male	346	55.987	Yes	198	32.039
Female	272	44.013	No	420	67.961
Age			Company size		
20–30	344	55.663	Less than 100 people	144	23.301
31–40	150	24.272	100–300 people	114	18.447
41–50	103	16.667	300–500 people	68	11.003
51–60	19	3.074	500–1,000 people	119	19.256
More than 60	2	0.324	More than 1,000 people	173	27.994
Education			Type of industry		
High school	175	28.317	Catering service	42	6.796
Junior college	148	23.948	Information service	40	6.472
Undergraduate	241	38.997	Electrical and Electronic	56	9.061
Master	54	8.738	Bio-manufacturing	10	1.618
Marital status			Financial service	33	5.340
Married	285	46.117	Educational services	48	7.767
Unmarried	317	51.294	Other industries	389	62.945
Divorced	16	2.589	Work online during pandemic		
Work years			Yes	371	60.032
Less than 1	85	13.754	No	247	39.968
1–5 years	256	41.424	Online working time per day		
6–10 years	113	18.285	Less than 1 h	113	18.285
11–15 years	61	9.871	1–3 h	171	27.670
16–20 years	46	7.443	3–5 h	121	19.579
More than 21	57	9.223	5–7 h	89	14.401
			More than 7 h	124	20.065

### Measures

Vineland Social Maturity Scale extensively used in foreign studies was adopted in this study, and experts and students majoring in English were invited to translate the scale for several times to ensure the accuracy of the language expression in the questionnaire. Likert five-point scale was generally used in the questionnaire except for items about personal background information, with 1 representing strongly disagree and 5 indicating strongly agree. Perceived organizational support adopted the scale revised by Akgunduz et al. (2018) that was based on the original scale developed by Eisenberger et al. (1986), and it includes a total of 8 measuring items, such as “The organization appreciates any extra effort from me” and “The organization would listen any complaint from me.” Occupational self-efficacy adopted the scale revised by Rigotti et al. (2008), and it was revised to integrate 6 items of higher reliability and validity, such as “I can remain calm when facing difficulties in my job because I can rely on my abilities” and “When I am confronted with a problem in my job, I can usually find several solutions.” Subjective wellbeing adopted the scale revised by Keyes (2005), which owns three measuring dimensions of emotional, psychological, and social wellbeing, as well as 12 measuring items, such as “In general, I consider myself very happy at work” and “Compared to most of my other colleagues, I consider myself happier.” Job performance adopted the scale revised by Chiang and Hsieh (2012) based on previous studies, with a total of 6 measuring items, such as “Fulfilling specific job responsibilities” and “Meeting performance standards and expectations.”

### Data Analysis Strategy

This study tested the hypotheses of the research framework and included paths via structural equation modeling. The hypotheses of the research framework are tested and paths are included in this study via structural equation modeling. We verified the reliability and validity using SPSS 23.0 and IBM-AMOS 23.0 before the construction of a structural model. In order to test the construct validity, confirmatory factor analysis (CFA) was performed using IBM-AMOS statistical program, v. 23.0 for Windows. Finally, partial least squares structural equation modeling (PLS-SEM) was adopted to construct the structural model; specifically, verification of the structural model was performed using SmartPLS 3.0 (path analysis).

## Results

### Measurement Model

Table 2 shows the results: Cronbach’s α scores are from 0.713 to 0.900, showing the high internal consistency of all constructs. Similarly, the combined reliabilities of all constructs are high, from 0.920 to 0.940. Moreover, we measured convergent validity and discriminant validity. The CRs of all constructs are above 0.7, and the AVEs are higher than 0.50 (Hua and Wang, 2019), showing sufficient convergent validity. Furthermore, to examine discriminant validity, we compared the square root of the AVE and the cross-correlations among the latent constructs (Li et al., 2018). The square root of AVE for each latent construct (see Table 2) is greater than its cross-correlation with other constructs, confirming discriminant validity.

**TABLE 2:** Measurement model.

	1	2	3	4	5	6
1.Organizational support						
2.Self-efficacy	0.446					
3.Emotional wellbeing	0.629	0.632				
4.Psychological wellbeing	0.595	0.689	0.832			
5.Social wellbeing	0.551	0.643	0.797	0.824		
6.Job performance	0.391	0.640	0.564	0.627	0.597	
Mean	3.380	3.971	3.796	3.829	3.928	3.883
SD	0.787	0.683	0.744	0.709	0.713	0.600
Cronbach’s α	0.919	0.898	0.903	0.894	0.913	0.907
AVE	0.675	0.831	0.776	0.760	0.794	0.682
CR	0.939	0.937	0.933	0.927	0.939	0.928x

To keep the study from being influence by severe common method biases, AMOS 23 was adopted in this study to test the bifactor model with method factors added on the basis of a 4-factor model (Podsakoff et al., 2003). As shown in Table 3, after method factors were added on the basis of the 4-factor model, CFI and TLI increased by 0.01 and did not exceed 0.1, RMSEA decreased by 0.008 and SRMR declined by 0.024, all of which did not exceed 0.05, indicating that there exists no significant change in the model fitting index, and there is no severe common method bias.

**TABLE 3:** Results of common method bias.

Model	cmin	cmin/df	CFI	TLI	RMSEA	SRMR
1-factor model	3760.545	22.121	0.629	0.585	0.185	0.125
2-factor model	2723.662	16.116	0.736	0.703	0.157	0.129
3-factor model	1690.444	10.122	0.842	0.821	0.122	0.111
4-factor model	444.579	2.761	0.971	0.965	0.053	0.047

#### Inner Model Analysis

Partial least squares structural equation modeling (PLS-SEM) was adopted to construct the structural model; specifically, verification of the structural model was performed using SmartPLS 3.0 (path analysis). According to Hair et al. (2017), this study assessed the *R*^2^, beta (β) and *t*-value. Their suggestions also emphasized the predictive relevance (*Q*^2^) as well as the effect sizes (*f*^2^). In the structural model, *R*^2^-values obtained for subjective wellbeing (*R*^2^ = 0.626) and job performance (*R*^2^ = 0.490) were larger than 0.3. Prior to hypotheses testing, the values of the variance inflation factor (VIF) were determined. The VIF values were less than 5, ranging from 1.257 to 2.673. Thus, there were no multicollinearity problems among the predictor latent variables (Hair et al., 2017).

**FIGURE 2:**
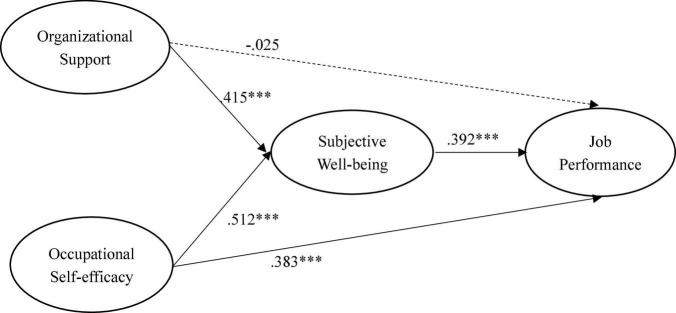
Results of structural model. *** of *p* < 0.001.

Figure 2 shows the results of the hypothesized relationships and standardized coefficients in inner model. The results showed that perceived organizational support (β = −0.025, *f*^2^ = 0.001, *p* > 0.1) was not significantly related to job performance, which not supporting H1. However, perceived organizational support (β = 0.415, *f*^2^ = 0.366, *p* < 0.001) was positively and significantly related to subjective wellbeing, supporting H2. In addition, our results found that subjective wellbeing (β = 0.392, *f*^2^ = 0113, *p* < 0.001) was positively and significantly related to job performance, supporting H3. The results found that occupational self-efficacy was positively and significantly related to subjective wellbeing (β = 0.512, *f*^2^ = 0.557, *p* < 0.001) and job performance (β = 0.383, *f*^2^ = 0.147, *p* < 0.001), supporting H5 and H6. The Stone-Geisser *Q*^2^-values obtained through the blindfolding procedures for subjective wellbeing (*Q*^2^ = 0.543) and job performance (*Q*^2^ = 0.325) were larger than zero, supporting the model has predictive relevance (Hair et al., 2017).

#### Examination of Mediating Effects

Subjective wellbeing in the structural model can be regarded as mediating variable. In order to understand whether subjective wellbeing has mediating effects, a bootstrapping procedure is further carried out on the structural model. Results displayed in Table 4 indicated that indirect effects of subjective wellbeing were supported, which supporting H4 and H7. It shows that the setting of mediating variable plays an important role in the structural model. In particular, subjective wellbeing, similar to the results of previous studies, can highlight the effects of antecedents in the model, forming strong positive psychology, which are then reflected in the outcome variables.

**TABLE 4:** Indirect effect of structural model.

Paths	Std. β	Std. error	*t*-value	Decision
Perceived organizational support→ Subjective wellbeing→ Job performance	0.162***	0.034	4.731	Support
Occupational self-efficacy→ Subjective wellbeing→ Job performance	0.201**	0.048	4.200	Support

** if p < 0.01; *** if p < 0.001.

## Conclusion and Discussion

### Discussion

Based on the conservation of resource theory, the study establishes a verifiable conceptual framework, and discusses the impact of internal and external resources on employees’ subjective wellbeing and job performance from the concept of internal and external resources. First, the findings show that perceived organizational support and occupational self-efficacy (H2 and H6), as external and internal resources, play a positive impact on subjective wellbeing in the context of the COVID-19 pandemic. These implied that the intrinsic psychological motivation arising from employees’ behaviors become more important in the positive psychology. The result is in concert with the previous studies that job resources involve physical, psychological, social, and organizational aspects (Harju et al., 2016). It cannot only assist employees in better implementing the work target, but also stimulate their personal growth and development. High internal and external resources provide employees with confidence in contending with work challenges and difficulties, and improve adaptation to the external high-risk environment and endow employees with more positive psychological quality, that is, to increase their subjective wellbeing.

Furthermore, the research findings show that, among antecedents in job performance, only occupational self-efficacy plays a positive and significant impact (H5). Such result is similar to that of previous studies (Hochwarter et al., 2006; Downes et al., 2021). Occupational self-efficacy is a kind of valuable individual resource for employees, which can increase the reserve of employees’ own psychological resources, improve the probability of getting the value-added spiral for employees, reduce the possibility of falling into the lost spiral, thus promoting the positive performance of employees and avoiding negative phenomena, such as job burnout or stress derived from resource depletion, as far as possible (Hobfoll, 2002), to improve job performance.

Research findings show that the relationship between perceived organizational support and job performance was insignificant (H1), which is different from previous arguments of scholars (Aselage and Eisenberger, 2003; Hurt et al., 2017). One of the possible reason is that perceived organizational support is a static resource supply, which is only the accumulation of static resources without being applied or integrated, and it fails to be reflected in actual job performance. As Watt and Hargis (2010) mentioned, perceived organizational support that changes depending on the impact of the organization and the external environment will affect employees’ interpretation of perceived organizational support and organizational motivation, and only by strengthening and integrating other factors can the impact on job performance be presented. Finally, the discussion on antecedent variables of job performance is expanded. At present, despite there are many domestic and foreign studies on job performance and various antecedent variables, there are few studies on antecedent variables of job performance from organizational and individual aspects. In addition, occupational self-efficacy is regarded as the mediating effect between perceived organizational support and job performance in most previous studies (Alisic and Wiese, 2020). In addition to occupational self-efficacy, research findings show that subjective wellbeing has positive and significant impact on job performance (H3). This is consistent with the arguments of Bryson et al. (2017); Darvishmotevali and Ali (2020), and Lee et al. (2021) that employees can ensure the fulfillment of tasks securely under uncertainties and high risks when they feel the intense wellbeing. This finding also implies that a positive psychological attitude significantly facilitates employees to improve their job performance.

Meanwhile, through testing the mediating effect, it is found that subjective wellbeing plays a complete mediating role among perceived organizational support, occupational self-efficacy, and job performance (H4 and H7). Perceived organizational support and occupational self-efficacy that employees own influence job performance via the meditating mechanism of subjective wellbeing. Such result is consistent with that from Watt and Hargis (2010) and Lee Lee et al. (2021). Perceived organizational support that employees feel is direct, and their judgment of occupational self-efficacy only stays at the superficial cognitive level, while the subjective wellbeing is the sublimation and further summary of the two. When employees feel wellbeing, they will usually achieve good job performance. It supports the early findings from scholars that wellbeing from work plays a mediating effect on job performance, which is one of the intrinsic psychological motivations affecting employees’ job performance (Lee et al., 2021). However, differing from the studies of Lent et al. (2016) and Meyers et al. (2019), this study also considers psychological effects of global environmental events, and enriches the theoretical model and mediating role of subjective wellbeing.

### Managerial Implications

First, organizations strive to provide employees with various kinds of support they need at work. Organizations should first show respect and emotional support to employees. Especially during crisis like the COVID-19 pandemic, humanized care from organizations can make employees feel warm, which contributes to enhancing their sense of belonging and making them work harder. Second, it is worth noting that enterprises necessarily provide employees with instrumental support when they are at work, especially when working at home, more convenient online office conditions provided by organizations are necessary, thus unnecessary work difficulties are reduced to ensure the accomplishment of performance.

Second, occupational self-efficacy, as a kind of occupational self-efficacy in a specific field, is not as stable as other personality traits (Caesens and Stinglhamber, 2014), and has certain plasticity. Thus, organizations can enhance the occupational self-efficacy of employees through persuasion, encouragement, training, and other ways. Administrators need to realize that, comparing with advanced technology, work experience or occupational skills, positive internal resources of employees often play a greater role in job performance. In view of this, enterprises should formulate reasonable strategies to enhance the occupational self-efficacy of employees, so as to improve their job performance. Besides, from the individual aspect, employees should cultivate their consciousness of occupational self-efficacy in daily work to increase their individual resources of personality traits, so that they are provided with enough resources when facing crisis events to keep job performance from being affected.

Finally, organizations should emphasize and strive to improve the subjective wellbeing of employees at work. The findings show that perceived organizational support affects job performance completely through subjective wellbeing, and occupational self-efficacy partly influences job performance through subjective wellbeing. Thereby, making employees own and maintain high subjective wellbeing is quite vital for improving job performance. Organizations can improve employees’ subjective wellbeing by providing employees with a good environment for growth, convenient work facilities, regular care, fair and equitable working regulation, and harmonious interpersonal relationships.

### Research Limitations and Suggestions for Future Studies

One disadvantage of this study lies in the questionnaire, and all of them are self-reported, which may have a certain homologous bias. Thus, a survey, in which a leader-employee pair is adopted, is suggested, so that the leader can evaluate the job performance of the employee. Moreover, static cross-sectional data are adopted in this study. Time series data can be obtained through follow-up surveys for subsequent studies, and a dynamic survey of perceived organizational support, occupational self-efficacy, subjective wellbeing, and job performance of employees during different periods, including the beginning, middle and end of the COVID-19 pandemic, can be conducted to compare the differences in various periods. In this study, we selected perceived organizational support and occupational self-efficacy as independent variables, and discusses the influence of subjective wellbeing on job performance from the organizational and individual aspects which are regarded as external and internal resources, respectively. Other researchers can also select other internal and external resources to explore the influence on job performance through other mediating variables from these two aspects.

In addition, the study has found an obvious correlation between perceived organizational support and occupational self-efficacy, but this relationship needs to be further clarified and verified. One explanation for the correlation is that employees with strong occupational self-efficacy have high self-confidence, sufficient internal individual resources and less dependence on the external surroundings, so they are more likely to show positive evaluation on organizational support and feel strong perceived organizational support. Another explanation shows that when employees feel organizational support, it is beneficial to the conservation of occupational self-efficacy, which is a kind of individual resource, so that their judgment of being able to accomplish work tasks is enhanced. Finally, the research context of this paper is the sudden outbreak of the COVID-19 pandemic, the influence of the pandemic outbreak tends to be extensive, which covers a wide range of fields. However, the paper does not select a specific industry for research, other scholars can choose a specific field for more detailed research, or choose to study the relationships among these variables in other contexts, so as to draw more general conclusions.

## Data Availability Statement

The raw data supporting the conclusions of this article will be made available by the authors, without undue reservation.

## Ethics Statement

The studies involving human participants were reviewed and approved by the University of Taipei. The patients/participants provided their written informed consent to participate in this study.

## Author Contributions

W-XZ contributed to the ideas of field research, design of research methods, and empirical analysis. LS, MZ, and MP contributed to the collection of data and data analysis. W-XZ and MP participated in developing the research design, writing, and interpreting the analysis. All authors contributed to the literature review and conclusion.

## Conflict of Interest

The authors declare that the research was conducted in the absence of any commercial or financial relationships that could be construed as a potential conflict of interest.

## Publisher’s Note

All claims expressed in this article are solely those of the authors and do not necessarily represent those of their affiliated organizations, or those of the publisher, the editors and the reviewers. Any product that may be evaluated in this article, or claim that may be made by its manufacturer, is not guaranteed or endorsed by the publisher.
